# Environmental heterogeneity explains coarse–scale β–diversity of terrestrial vertebrates in Mexico

**DOI:** 10.1371/journal.pone.0210890

**Published:** 2019-01-25

**Authors:** Pilar Rodríguez, Leticia M. Ochoa–Ochoa, Mariana Munguía, Víctor Sánchez–Cordero, Adolfo G. Navarro–Sigüenza, Oscar A. Flores–Villela, Miguel Nakamura

**Affiliations:** 1 Comisión Nacional para el Conocimiento y Uso de la Biodiversidad, Mexico City, Mexico; 2 Museo de Zoología, Facultad de Ciencias, Universidad Nacional Autónoma de México, Mexico City, Mexico; 3 Instituto de Biología, Universidad Nacional Autónoma de México, Mexico City, Mexico; 4 Centro de Investigación en Matemáticas, Asociación Ci, Guanajuato, Mexico; Tuscia University of Viterbo, ITALY

## Abstract

We explored the hypothesis that high β–diversity of terrestrial vertebrates of Mexico is associated with a high environmental heterogeneity (HEH) and identify the drivers of β–diversity at different spatial scales. We used distribution range maps of 2,513 species of amphibians, reptiles, mammals, and birds occurring in Mexico. We estimated β–diversity for each taxon at four spatial scales (grid cells of 2°, 1°, 0.5° and 0.25°) using the multiplicative formula of Whittaker β_w_. For each spatial scale, we derived 10 variables of environmental heterogeneity among cells based on raw data of temperature, precipitation, elevation, vegetation and soil. We applied conditional autoregressive models (CAR) to identify the drivers of β–diversity for each taxon at each spatial scale. CARs increased in explanatory power from fine–to–coarse spatial scales in amphibians, reptiles and mammals. The heterogeneity in precipitation including both, coefficient of variation (CV) and range of values (ROV), resulted in the most important drivers of β–diversity of amphibians; the heterogeneity in temperature (CV) and elevation (ROV) were the most important drivers of β–diversity for reptiles; the heterogeneity in temperature (ROV) resulted in the most important driver in β–diversity for mammals. For birds, CARs resulted significant at fine scales (grid cells of 0.5° and 0.25°), and the precipitation (ROV and CV), temperature (ROV), and vegetation (*H*) and soil (*H*) were heterogeneity variables retained in the model. We found support for the hypothesis of environmental heterogeneity (HEH) for terrestrial vertebrates at coarse scales (grid cell of 2°). Different variables of heterogeneity, mainly abiotic, were significant for each taxon, reflecting physiological differences among terrestrial vertebrate groups. Our study revealed the importance of mountain areas in the geographic patterns of β–diversity of terrestrial vertebrates in Mexico. At a coarse scale, specific variables of heterogeneity can be used as a proxy of β–diversity for amphibians and reptiles.

## Introduction

The change in species composition between sites, known as β–diversity [1], is a critical component of biodiversity. The study of β–diversity can provide useful insights into the ecological and evolutionary processes maintaining biodiversity [2], the changes of biodiversity along environmental gradients [3], and human–induced impacts and response to climate change [4]. Moreover, β–diversity also provides information for the delineation of biotic regions or biotic transitions, and the mechanisms through which regional biotas are constituted [5]. The studies of geographic patterns of β–diversity at macroecological scales, particularly the terrestrial vertebrates are more frequent, due to the availability of worldwide information [3,6,7]. The interest in the study of β–diversity at macroecological scales, and in the processes underlying the geographic patterns of β–diversity, are increasing dramatically. β–diversity is determined through a complex array of processes that reflect the interaction of species (i.e., vagility, environmental tolerances, resource use, and reproductive strategies) with characteristics of the physical environment (i.e., environmental dissimilarity, physical distance, and isolation) over ecological and evolutionary time [6]. Understanding which factors drive β–diversity is biologically relevant, and most studies address the effect of the environmental variables and geographic scale on β–diversity [3,6–9], as well as historical factors on β–diversity [10–13]. The three mechanisms summarized by Stein and Kraft [14] to explain how environmental heterogeneity promote species diversity might be applied to the relationship environmental heterogeneity– β–diversity. In turn, an increase in environmental gradients and in the amount of habitat types, resources and structural complexity should increase the available niche space [ecological space] with more species coexisting as consequence. Second, environmentally heterogeneous areas are more likely to provide shelter and refuges from adverse environmental conditions and periods of climate change, allowing the persistence of species with different requirements. Finally, the probability of speciation events resulting from isolation or adaptation to diverse environmental conditions should increase with higher.

The megadiversity of mammals in Mexico is the result of the combination of moderate α–diversity and high β–diversity associated, in turn, with the high number of species of small distributional range [15,16]. Furthermore, several trends of β–diversity have been recognized at a macroecological scale for Mexican terrestrial vertebrates. For example, β–diversity is higher in taxa with low vagility, amphibians and reptiles, and lower in taxa of high dispersal ability [17,18]; is higher in the central and southeast Mexico, and lowest in the Yucatán Peninsula [18,19], and varies with the spatial scale, being higher at coarse large scales (i.e., 2° x 2° cell size), particularly in amphibians and reptiles [18]. Despite the importance of β–diversity for understanding the high biodiversity of terrestrial vertebrates in Mexico, the factors explaining β–diversity patterns remain poorly explored [20]. The complex geological history, which promoted the predominance of endemic and small distributional ranges species [21], and the convergence in the country of faunas from different historical sources (Nearctic and Neotropical realms) are the two most frequently invoked hypotheses to explain the extraordinary number of species and the high β−diversity in the region [22]. Complementarily, proximal explanations postulate that the high β–diversity is the result of the current environmental and climatic complexity of Mexico [17,19,23]. Herein, we aim to explore the hypothesis that β–diversity is related to the high environmental heterogeneity (HEH) of Mexico. We used four groups of terrestrial vertebrate, amphibians, reptiles, mammals and bird, because the patterns of species richness, endemism and range size at macroecological scales are well known. Specifically, we addressed (1) if current geographical patterns of β–diversity are associated to the environmental heterogeneity; (2) to identify the drivers of β–diversity associated to each group, and (3) to identify the drivers of β–diversity at different spatial scales. Regarding the second question, our analysis was guided for the following hypothesis: The importance of different variables of environmental heterogeneity differs among taxa due to the differences in physiology and vagility. For example, amphibians and reptiles that are ectotherms and present low vagility should be affected more strongly by proxies of temperature and precipitation than mammals and birds, that are endotherms and have high dispersion ability. Regarding the thirst question, our analysis was guided by the following hypothesis: (1) we expect that the drivers of β–diversity vary at the different scales, as previous work has demonstrated that spatial patterns of β–diversity vary with the spatial scale [18,24]. Moreover, the effect of the environmental heterogeneity should increase with spatial scale, because larger sampling units generally include greater variability in environmental conditions.

## Materials and methods

### Distribution range maps

We used a comprehensive database of terrestrial vertebrates in Mexico, comprising published distribution range maps of 364 amphibians and 811 reptiles (96.8% of 376 and 93.8% of 864 respectively [25,26], 344 mammals (76% of 451) [27], and 883 resident birds (81% of 1,085) [28]. The distribution range maps are based on models of the ecological niche of the species. These sets of distribution maps (about 1 km^2^ of resolution) have been widely used in analyses of biogeography and conservation of the Mexican fauna (for example [16,18,29]), and most maps are openly available at http://www.conabio.gob.mx/informacion/gis/.

### Measuring β–diversity

To analyze β–diversity at different spatial scales, we followed the conceptual framework proposed by Barton and colleagues [30], who define the ‘spatial windows’ of the study considering both elements of spatial scale: grain and extent. We defined the spatial window of our study by maintaining the grain fixed (1 km^2^, which is the pixel size), and varying the extent. To do that, we divided the Mexican territory into grid cells of four sizes, 2°x2° (~200×200 km), 1°x1° (~100×100 km), 0.5°x0.5° (~50×50 km), and 0.25°x0.25° (~25×25 km).

To estimate β–diversity, we used the multiplicative formula $\beta w=\gamma /\bar{\alpha }$ [1], where γ is gamma diversity, i.e. the number of species in a *region*, and $\bar{\alpha }$ is alpha diversity, the average number of species of the *sites* that conform the region. In this study, a *region* is one of the cells in which the country was subdivided. For example, at the coarse scale (2° x 2° grid cells), there are around 30 regions nationwide. The *sites* included all the pixels of 1 km^2^ from each region. A region of 2° includes approximately 40,000 pixels. We used βw because is methodologically independent of alpha and gamma diversity [31], allowing the comparison between taxa of different number of species. β–diversity was estimated per group at each spatial scale. It is important to note that we did not compared β–diversity between scales but among groups at the same scale. β–diversity includes values from 1, in which all species occupy all the pixels of the cell (the extreme case of low β–diversity), to γ–diversity, in which each species of the region occurs in only one of the sites (the extreme case of highest β–diversity). Borderline and coastal grid–cells were eliminated when more than 50% of the unit area occurred outside Mexico or in the sea.

### Variables of environmental heterogeneity

We included the environmental variables most frequently associated to β–diversity of terrestrial vertebrates, such as precipitation, temperature, and elevation [3,6–9]. From the raw data, we derived two types of variables that are proxies of the heterogeneity of the precipitation, temperature, and elevation among regions: the range of values ROV, and the coefficient of variation CV. The range of values ROV is the range of raw data values within that region: maximums minus minimums [6,14]. The coefficient of variation CV is the ratio of standard deviation to the mean [14]. A low CV indicated that the values of the variables are relatively similar in the region, and a high CV indicated that few sites of the region show extreme values (Table 1). The annual precipitation, and the maximum and minimum temperatures data were obtained from Worldclim Project [32] at 30 arc–seconds (~ 1 km^2^). The elevation data were obtained from digital elevation model [33] at around 30 m^2^. Additionally, we introduced two proxies of environmental heterogeneity less frequently used in the analyses of β–diversity: vegetation type, obtained from the potential primary vegetation map of Mexico [34], and soil types, obtained from the map of soils of Mexico [35]. From the raw data, we calculated the Shannon–Wiener index (*H*) [7,8]. To calculate *H* for vegetation types, we computed the area of the region (in km^2^) covered by the different vegetation types. *H* takes values from zero, when one vegetation type occupies the entire region (and then the heterogeneity is low), to the logarithm of *S* (*S* = number of vegetation or soil types), when the vegetation types are equally distributed among the region (and then the heterogeneity is high). The same procedure was repeated to obtain *H* of soil types (Table 1). All the environmental variables were generated for each spatial scale and mapped (Fig A in S1 Appendix).

**Table 1:** Proxies of environmental heterogeneity included in the analyses. For each spatial scale, we derived 10 variables of environmental heterogeneity among cells based on raw data of temperature, precipitation, elevation, vegetation and soil: the range of values ROV, and the coefficient of variation CV. For vegetation and soil types, we calculated the Shannon–Wiener index (H).

Range of elevation	ROV Elev = max(Elev) − min(Elev)
Range of precipitation	ROV Pp = max(Pp) − min(Pp)
Range of temperature	ROV Tm = max(Tmax) − min(Tmin)
Coefficient of variation of elevation	CV Elev = Stdv(Elev) ∕ mean(Elev)
Coefficient of variation of precipitation	CV Pp = Stdv(Pp) ∕ mean(Pp)
Coefficient of variation of temperature	CV Tm = Stdv(MAT) ∕ mean(MAT), where MAT = mean annual temperature.
Shannon–Wiener diversity index of vegetation, from a shapefile containing nine vegetation types.	$Veg(H)=-\sum_{i=0}^{n}p_{i}ln\;p_{i}$, where *p*_*i*_ is the proportion in area (km^2^) occupied by each vegetation type (*i*) within the grid cell.
Shannon–Wiener diversity index of soil, from a shapefile containing 25 soil types.	$Soil(H)=-\sum_{i=0}^{n}p_{i}ln\;p_{i}$, where *p*_*i*_ is the proportion in area (km^2^) occupied by each soil type (*i*) within the grid cell.

### Testing the hypothesis of environmental heterogeneity (HEH)

To examine the HEH, we used conditional autoregressive (CAR) models, an approach which considers spatial autocorrelation [36]. These individual models—separate ones formulated for each spatial scale and taxa—assume that mean β–diversity is a linear function of the set of environmental heterogeneity variables. In contrast to ordinary linear regression, random errors about the mean in CAR models are not assumed to be independent. Instead, an explicit spatial dependence is allowed, postulated in terms of a neighborhood structure that amounts to specify exactly which additional cells (regions) are deemed to influence a given cell (region). In addition to the standard linear coefficients of predictor variables, an additional parameter *λ* is incorporated [37], which measures the impact of the spatial structure (where *λ* = 0 means that error terms are in fact, independent, so that no spatial structure is relevant). Our numerical implementations were performed in R [38], using package spdep [39], and specifically, function *spautolm* [37], which estimates parameters by normal maximum likelihood.

Before fitting autoregressive models, we standardized the values of the ranges of each variable to proportional values from 0 to 1, selecting the maximum value of the range and then divided the rest by that value. The variables were tested for collinearity (the degree of spatial correlation between two variables, above 0.8 of correlation coefficient were considered collinear); if two collinear variables were found, we discarded one to avoid overfitting. In all spatial scales, elevation (ROV) was collinear with the temperature (CV) and was discarded. At 2° scale, the precipitation (ROV) and (CV) were collinear, and discarded CV (Table A in S1 Appendix and S2 Appendix for raw data).

To comply with CAR model normality assumptions, a Box–Cox transformation for β–diversities was specified (not shown), via the power constant [40]. All Box–Cox powers were less than one, because the distribution of β–diversities was typically right–skewed and called for concave transformations to induce normality. Finally, the spatial structure (argument *listw* in function *spautolm*) was created via the neighborhood matrices using *Queen contiguity* for each scale produced by geoda 1.4.0 [41].

A second, important model assumption regards variances. The standard assumption for maximum likelihood estimation is that variances for each data point are constant. However, there are clear grounds for expecting non–homogeneous variances in the β–diversities, due simply to the fact that the denominator of each β–diversity is an average obtained with differing numbers of species. For this reason, we considered it important to enable a weighting scheme in the CAR models [37], defined by setting the weights argument in function *spautolm* to be equal to the reciprocal of the number of species. The inclusion of this weighting scheme does not amount to adding richness as a new independent variable. Its function is cautionary, merely to inform the maximum likelihood procedure that variances may not be homogeneous so that parameter estimates are theoretically more precise and the ensuing *P*–values conceptually correct. The idea is that if a β–diversity is potentially imprecise because of a small species richness, that data point’s influence on parameter estimation in the CAR model is down-weighted in relation to all data points used in the fit.

In summary, a combination of transformation and weighting techniques was called upon to promote basic technical assumptions. Once put into effect, model validation included inspection of residuals to verify normality. Residuals were also tested for any remaining spatial autocorrelation using a Monte Carlo test for Moran’s *I* with 999 permutations (also implemented in package spdep), to verify that spatial effects were adequately accounted for by the given neighborhood structure.

## Results

**Fig 1:**
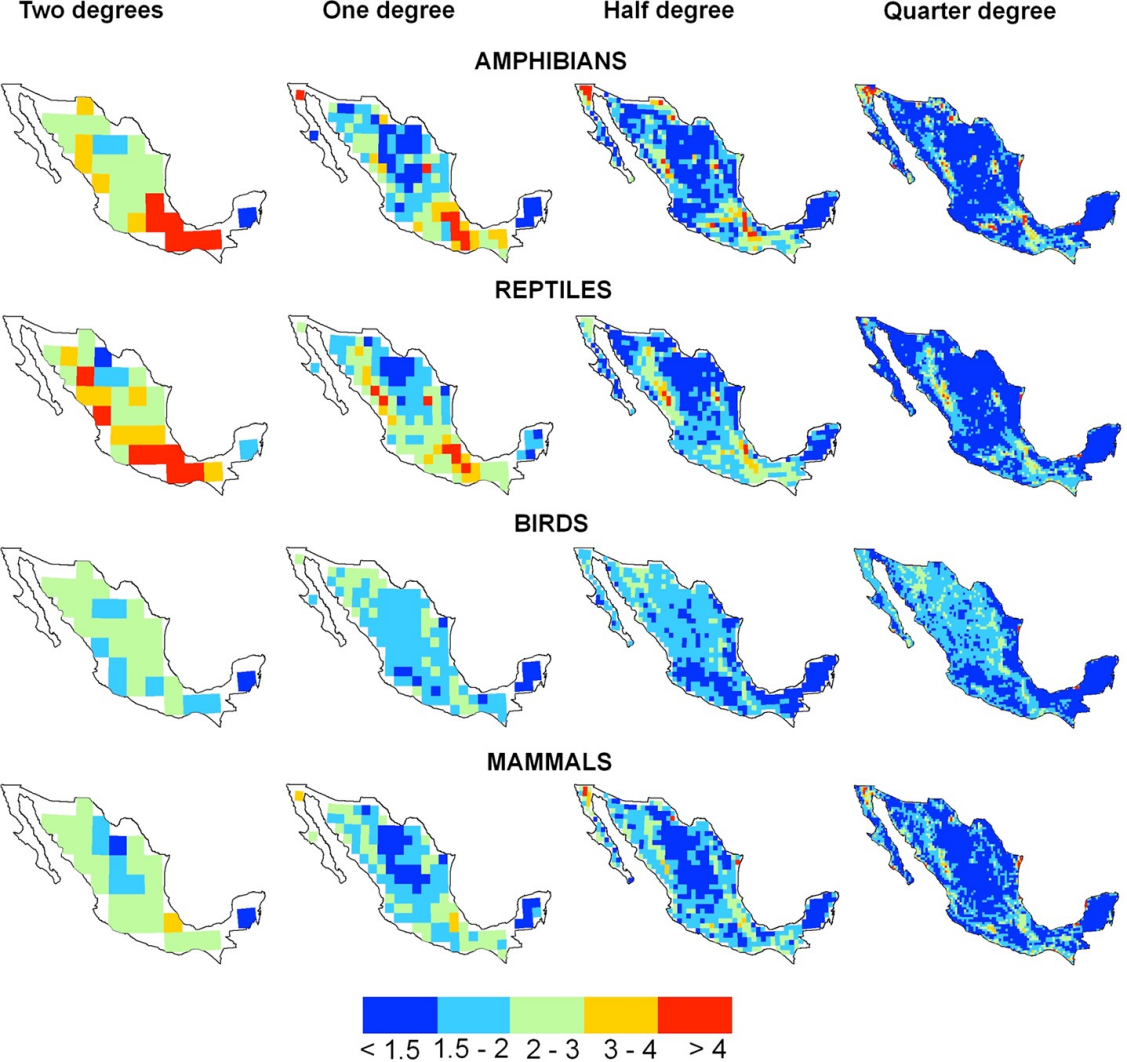
Geographic patterns of β–diversity for terrestrial vertebrates at different spatial scales in Mexico. The maps represent the spatial pattern of β–diversity by taxa and at different spatial scales. β–diversity was calculated with the multiplicative formula proposed by Whittaker $\beta w=\gamma /\bar{\alpha }$ [1], where γ is gamma diversity (the number of species in a *region*) and α_mean_ is alpha diversity (the average number of species of the *sites* that conform the region). A *region* is one of the cells in which the country was subdivided, and the *sites* included all the pixels of 1 km^2^ from each region.

The geographic patterns of β–diversity among amphibians and reptiles showed similar trends at coarse spatial scale (grid cells of 2°): higher at southern Mexico (an extremely complex region in terms of climatic and topographic conditions, with elevations that varies from the sea level to more than 4,000 m a.s.l.), and intermediate along the Sierra Madre Oriental (also, an extremely complex region in terms of climatic and topographic conditions). At smaller scales (grid cells of 1°, 0.5° and 0.25°), the geographic patterns of β–diversity among mammals, amphibians, and reptiles showed similar trends: higher in mountain ranges, mainly at the south of the Sierra Madre Oriental, and north of the Sierra Madre Occidental. Conversely, at all spatial scales, birds showed from intermediate to low β–diversity, in a significant portion nationwide (Fig 1).

**Fig 2:**
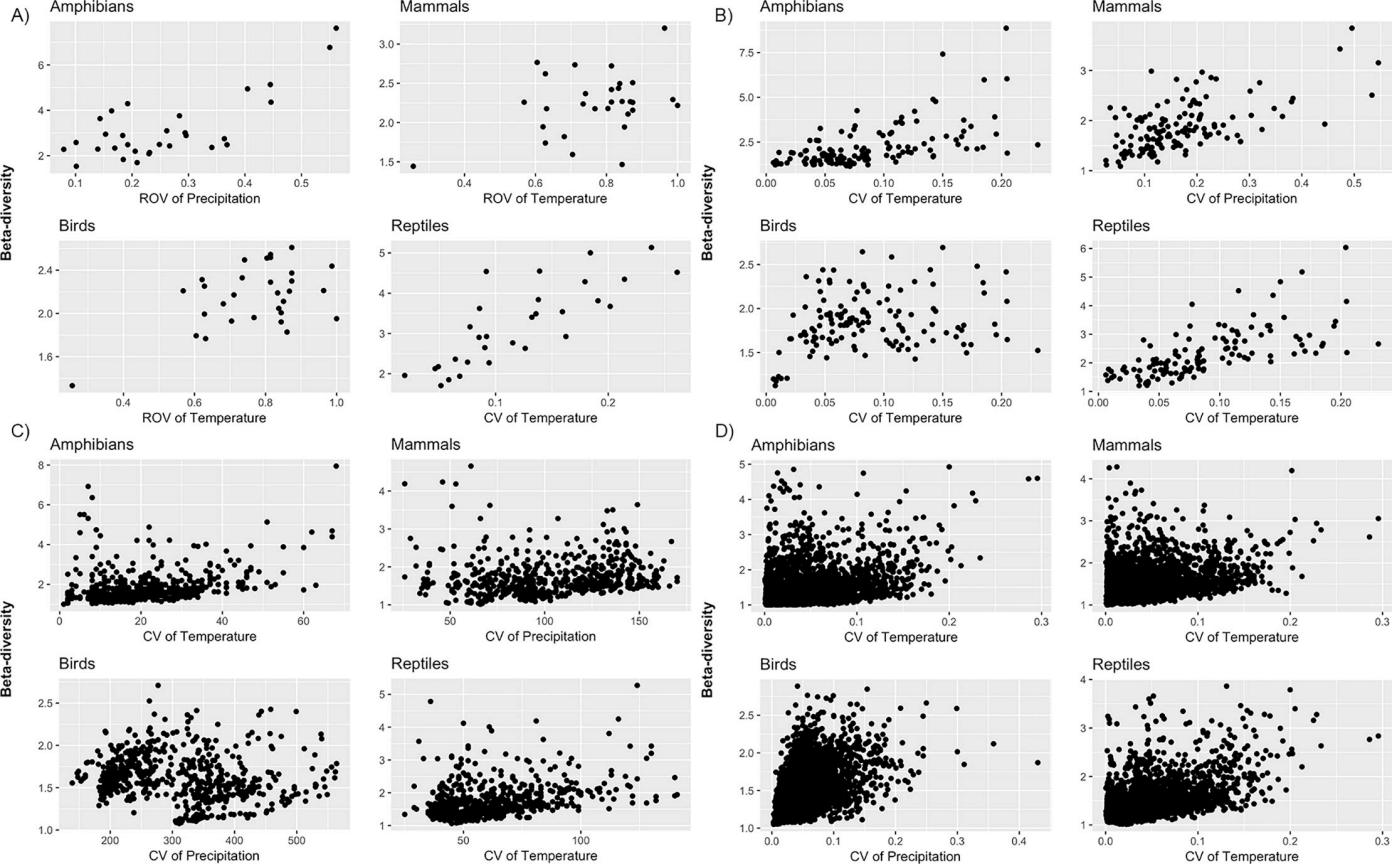
Scatterplots of β-diversity and the most significant variables of environmental heterogeneity. Scatterplots of β–diversity and the most significant proxies of environmental heterogeneity of A) amphibians; B) reptiles; C) mammals; and D) birds.

CARs increased in explanatory power from fine–to–coarse spatial scales in amphibians, reptiles and mammals. At coarse scale (grid cells of 2°), the models explained the 64% of the variation of β–diversity in amphibians and mammals, and the 79% in reptiles. The most important explanatory variables of environmental heterogeneity were different for each taxon: precipitation (ROV) for amphibians; temperature (ROV) for mammals; and elevation (ROV) for reptiles. In birds, CARs explained more than 70% of the variation of β diversity at the finest scale (grid cells of 0.25°), and the proxies of heterogeneity of precipitation (ROV and CV), temperature (ROV), vegetation (*H*) and soil (*H*) were significant (Fig 2 and Table 2).

**Table 2:** Results of the conditional autoregressive models fitted to explain the β–diversity of terrestrial vertebrates in Mexico.

Group	Scale	N	R^2^	Lambda	Intercept	CV Tm	ROV Pp	CV Pp	ROV Tm	CV Elev	Veg (H)	Soil (H)
	2°	30	0.64	–0.003	1.64	3.88	4.09***	–	0.81	–0.55	0.09	–0.52
	1°	127	0.60	–0.019	0.36***	1.19**	0.12	0.24	–0.09	–0.01	–0.04	–0.07*
**Amphibians **	0.5°	594	0.42	0.99***	0.04	–0.91**	–0.53***	0.69***	0.24**	0.02	–0.01	–0.05**
	0.25°	2770	0.47	0.96***	0.34***	0.79***	–0.16*	0.24*	–0.17***	0.06***	0.002***	0.00
	2°	30	0.79	0.005	1.82**	11.10***	1.27*	–	–0.56	–0.24	0.20	–0.03
	1°	127	0.60	–0.019	0.36***	1.66***	0.12	0.09	–0.12*	0.04	0.02	–0.03
**Reptiles **	0.5°	594	0.59	0.91***	0.37***	0.47***	–0.03	0.09	–0.14***	–0.07***	–0.002	–0.008
	0.25°	2770	0.66	0.98***	0.26***	0.49***	–0.05*	0.025	–0.098***	0.004	0.0004**	–0.008**
	2°	30	0.36	–0.007	1.32***	–1.94	0.24	–	1.42***	–0.14	0.09	–0.09
	1°	127	0.59	–0.013	0.15***	–0.49**	–0.04	–0.35***	0.36***	–0.04	0.04**	–0.002
**Birds**	0.5°	594	0.67	0.93***	0.31***	0.08	–0.24***	0.47***	0.12**	–0.06***	–0.002	–0.013
	0.25°	2770	0.73	0.96***	0.27***	0.25**	–0.35***	0.67***	0.22***	–0.05***	0.001***	–0.02***
	2°	30	0.64	–0.004	0.63	–1.06	0.52	–	1.33**	0.24	0.30	0.04
	1°	127	0.60	0.52*	0.17***	0.09	–0.004	0.34**	0.21**	0.09***	0.03	–0.04*
**Mammals **	0.5°	594	0.51	0.95***	0.27***	–0.01	–0.01	0.19**	–0.03	0.008	0.001	0.005
	0.25°	2770	0.49	0.89***	0.23***	0.33***	0.016	0.03	–0.002	0.05***	0.002***	0.02***

The '–' indicates variables eliminated in the pre–processing stage due to collinearity. Highlighted in grey are the most important variables in the model. The regression coefficient presented is the Nagelkerke pseudo–R–squared for the autoregressive models. All entries are estimated values of the corresponding parameter in the model, with levels of significance signaled as follows

* < 0.05

** < 0.01

*** < 0.001. All models were performed in R [38].

We found no spatial structure at 2° scale for any taxa. At 1° and 0.5° scales, reptiles were the only group that did not present spatial structure. At 0.25° scale, the data of all taxa showed significant spatial structure (Table 2). In every case, the value of Moran’s *I* test statistic was very low and non–significant for the residuals of the CAR models.

## Discussion

### Geographic patterns and drivers of β–diversity for amphibians and reptiles

At coarse spatial scale (grid cells of 2°), amphibians and reptiles showed high β–diversity in mountain elevations, mainly the Sierra Madre Oriental, and the Sierra Madre Occidental. These patterns coincided with that reported in other studies from Mexico [16,42,43] but not with that reported at continental level, where β–diversity is more uniform along the Mexican territory [3]. Interestingly, similarities in the geographic patterns between amphibians and reptiles were related to different variables of environmental heterogeneity associated to β–diversity. Amphibian and reptile β–diversities were associated to the heterogeneity of the precipitation (CV) and to the heterogeneity of the temperature (CV), respectively. Physiological differences between both taxa might explain these trends. Amphibians require water for reproduction, and the adults require environmental humidity and cooler temperatures for surviving [44]. Greater spatial variation in precipitation within a region (grid cells of 2°, that is, around 40,000 km^2^), indicates a greater range of local conditions, and thus, the possibility of numerous amphibian species inhabiting in a restricted subset of the region. For example, Los Tuxtlas, mountain range in southeastern Mexico, which showed the highest β–diversity at all the spatial scales (Fig 1), holds suitable humid tropical forest habitat for three amphibian species (*Pseudoeurycea lineola*, *P*. *orchimelas* and *P*. *werleri*). Similarly, all genera of endemic salamanders (*Parvimolge* and *Pseudoeurycea*), are distributed essentially at high elevation in Los Tuxtlas mountain range.

Reptile β–diversity was associated to the heterogeneity of the temperature (CV) and their correlated elevation (ROV). It is well documented that at the intermediate elevations, in short distances, important differences in temperature occurred. Most reptile species, due to its ectothermic condition, appear to be sensitive to rapid temperature ranges along elevational gradients. This might explain the high β–diversity of the reptiles along the mountain regions of Mexico. For example, along the Mexican Transvolcanic Belt and the Sierra Madre del Sur, which showed the highest β–diversity at the larger spatial scale (grid cells of 2°), it is possible to clearly distinguish regions, determined by endemic species of reptiles [42]. As far as we know, this is the first analysis of β–diversity of reptiles performed at macroecological scales, then it is no possible to compare our results.

The lack of association between variables of environmental heterogeneity and β–diversity in reptiles and amphibians at the finer spatial scales (grid cells of 0.5° and 0.25°) suggests that other variables not included in our study might play an important role. For example, at the landscape level, β–diversity of amphibians of Andalusia, Spain, was explained by the CV in mean annual NDVA, a proxy of energy available [45]. In the same study, β–diversity of the reptiles was explained by the CV in mean monthly NDVI and by the mean annual NDVI. The relationship between productivity and β–diversity is as follow: an increase in productivity is expected to promote an increase in habitat specialization. Habitat specialization increases the dissimilarity in species composition among places with the consequence increasing in β–diversity. Also, higher energy systems promote shorter generation times, which a consequent increasing in biotic interactions. The intensification of the biotic interactions might likely contribute to high species replacement due, for example to processes of competence [46]. Although these mechanisms are observed at smaller scales than our study (i.e. landscape), the effect might be perceived at macroecological scales (i.e. our grid cell of 0.25°). Thus, further studies aimed to understand the effect of environmental heterogeneity in β–diversity of Mexican vertebrates at macroecological scales should include proxies of productivity as environmental heterogeneity variables.

### Geographic patterns and drivers of β–diversity of mammals

Overall, the geographic pattern of β–diversity in mammals resulted similar to amphibians and reptiles, although subtle differences were observed. At coarse spatial scale (grid cells of 2°), β–diversity in mammals ranked intermediate to low nationwide, and the highest values was observed in complex and climatically heterogeneous southeastern Mexico. This pattern was also reported by studies performed at continental scale [6,9,47]. The southeastern Mexico holds a disproportionate number of endemic mammals, as is the case for amphibians and reptiles [16]. The proxy of heterogeneity associated to β–diversity in mammals was the temperature (ROV), contrasting to the above cited studies, that found that elevation was the main driver of β–diversity of mammals [6,9]. Our result is particularly intriguing, since mammals are able to cope with temperature variation compared to amphibians and reptiles. Our expectation was to observe a higher distribution overlapping in mammal species, and consequently low β–diversity in regions where the variations in temperature are high. It is unlikely that variation in temperature *per se*, directly affects β–diversity in mammals; rather, this variable may act as a surrogate of habitat differentiation [9]. Extreme values of temperature among a region of around 2,000 km^2^ might promote the existence of different habitat types, which are available for different species of mammals. For example, in southeastern Mexico, which showed high β–diversity, numerous species of small mammals, as *Rheomys mexicanus*, *Peromyscus yucatanicus*, *Microtus chinanteco*, *Ototylomys phillotis*, *Peromyscus furvus*, and *Sorex slateri*, limit their distribution range to specific habitats in restricted areas.

Our study differed with others that observed a high β–diversity of mammals in the Mexican Transvolcanic Belt [9,18,19], a geological and topographical complex region situated in central Mexico. We believe that these discrepancies are an effect of the difference of the spatial scales used in both studies. The extent of our analysis (grid cells of 2°) maybe is not large enough to capture the replacement of mammal species occurring in the Mexican Transvolcanic Belt, where different assemblages of mammals have been associated with a complex biogeographic history, and a high environmental heterogeneity [29]. In addition to differences in spatial scales, our study included species’ distributional ranges derived from ecological niche modeling, likely overestimating the actual range of species distributions [20,24]. Thus, differences in methodological approaches (spatial scales, and species range distributions) can affect the results of β–diversity patterns in mammals.

### Geographic patterns and drivers of β–diversity in birds

The geographic patterns of β–diversity in birds differed from other taxa. Overall, birds showed a low β–diversity nationwide. On average, birds showed larger species distribution ranges than the other taxa [16], then, the results were in agreement with our expectations, but also with other studies performed at global [3], continental [12] and at national levels [18]. However, our results disagree with studies performed at continental and global scales, which showed a high β–diversity of birds in higher elevation along the sierras of Mexico [6,7,9]. The discrepancies in the geographical patterns might be also attributable to methodological differences, as occurred with mammals, but in a more notorious way due to larger mean distributional ranges of birds [16].

Regarding the HEH in birds, the only significant model derived from the CARs analyses resulted at the finest spatial scale (grid cells of 0.5° and 0.25°), and a large number of variables of environmental heterogeneity were retained in the model (Table 1). The heterogeneity in the precipitation (CV) was the most important driver of β–diversity, contrasting to other studies performed with birds and mammals at a continental scales, for example, that found that the heterogeneity of the elevation resulted in the main drive of β–diversity of birds [9].

The lack of association of our environmental heterogeneity variables and the β– diversity patterns of birds at coarser spatial scales (grid cells of 2° and 1°) suggests that other processes operating at large geographical and historical scales not considered here might also be affecting. For example, in order to analyze the effect of the last glaciation events in the current patterns of β–diversity of birds at the continental scale, our colleagues [12] separated the two components of β–diversity: *turnover* (differences in composition caused by species replacement) and the *nestedness* (differences in species composition causes by species losses). The dominance of the component turnover over the component nestedness corroborated the importance of *in situ* processes of speciation–extinction on current patterns of β–diversity in Mexico. This pattern is characteristic of the regions of relatively stable climatic conditions for long periods, in contraposition with the footprint of the glaciation events, which creates the classical nesting pattern resulting from the process of extinction and re-colonization: species found in depauperated regions are subsets of species richer regions. In fact, the dominance of the turnover over the nestedness also reported for mammals [12], amphibians [11,12], and freshwater fishes [10], explains, in part, the high endemism and the predominance of small distributional range of species of Mexico [16], which determines the β–diversity patterns.

Our study revealed the importance of mountain areas in the geographic patterns of β–diversity of terrestrial vertebrates in Mexico. The high β–diversity implies that historical processes of isolation created divergence in species composition among these regions [9,20]. The variation in species composition among sites can be interpreted as resulting differentiation in species pool, in which the underlying basis is the geographical differentiation. Therefore, differences in the altitude seems to be the most appropriate proxy for the divergence in species composition in Mexico [14].

The spatial scale of our analyses, both in extent and grain, allowed to incorporate the effect of the environmental heterogeneity in different taxa. The current environmental heterogeneity explained the patterns of β–diversity of Mexico at coarse spatial scale (grid cells of 2°) in mammals, reptiles and amphibians, and at fine spatial scale (grid cells of 0.25°) in birds. The potential use of these findings in biodiversity conservation is relevant. It is possible to use specific variables of environmental heterogeneity as predictors of β–diversity, and as proxies of β–diversity. For example, the heterogeneity in the temperature (CV) resulted a good proxy of the spatial pattern of β–diversity of reptiles at the coarsest spatial scale (grid cells of 2°). In this sense, in regions poorly surveyed with high heterogeneity in temperature (CV), β–diversity of reptiles is expected to be high. Our study can also contribute to understand the potential impacts of climate change. At coarser spatial scales, changes in climate variability can affect the spatial patterns of β diversity, especially for species restricted to specific habitats and limited dispersal abilities.

## Supporting information

S1 Appendix Supporting information file containing Figure A and Table B. (DOCX)

S2 Appendix Supporting information file containing the data for the analyses performed. (ZIP)
