# Supplementary material for: Environmental heterogeneity explains coarse–scale β–diversity of terrestrial vertebrates in Mexico
Source: PLoS One. 2019 Jan 25;14(1):e0210890. doi: 10.1371/journal.pone.0210890 (PMC6347424; doi:10.1371/journal.pone.0210890)
Supplement: S1 Appendix — (DOCX) [file pone.0210890.s001.docx]

**S1 Appendix**

**Fig A. Geographic patterns of variables of environmental heterogeneity at different spatial scales.** Range was calculated, maximum minus minimum, within each cell. Coefficient of variation was calculated as the ratio of the standard deviation to the mean. Annual precipitation, and the maximum and minimum temperatures data were obtained from Worldclim Project [1] at 30 arc–seconds (~ 1 km^2^). The elevation data were obtained from digital elevation model [2] at around 30 m^2^. Vegetation type, obtained from the potential primary vegetation map of Mexico [3], and soil types, obtained from the map of soils of Mexico [4]. The scale of values was determined by Jenks natural breaks classification.


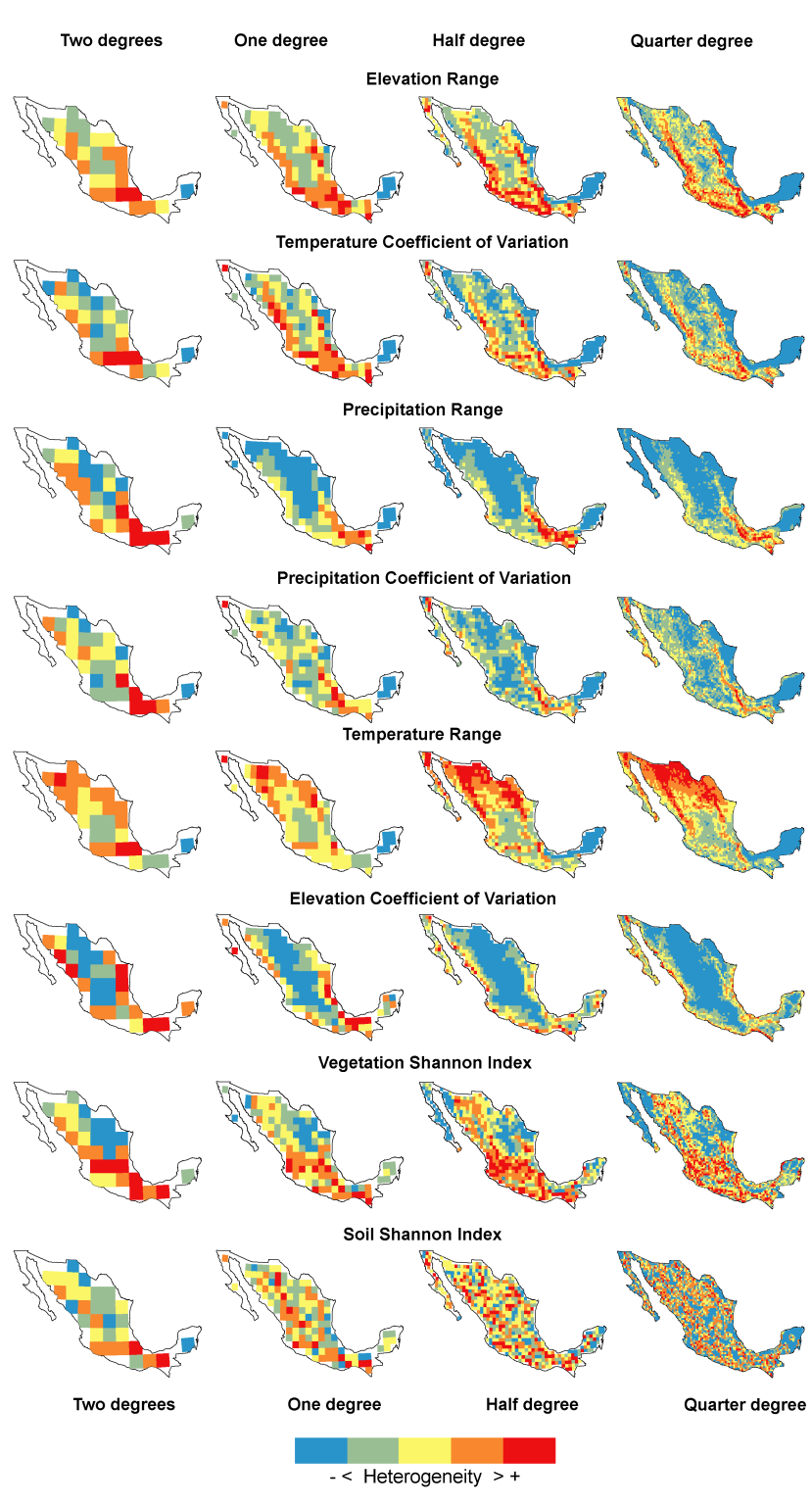


**Table A. Pair-wise correlations to test co-linearity among climatic environmental heterogeneity metrics.** Range of elevation (**ROV of Elev**), range of (**ROV of Pp**) range of temperature (**ROV of Tm**), coefficient of variation of elevation (**CV of Elev**), coefficient of variation of (**CV of Pp**) coefficient of variation of (**CV of Tm**). High correlation values are highlighted in grey.

| **Two degrees** | ROV of Elev | ROV of Pp | ROV of Tm | CV of Pp | CV of Elev | CV of Tm |
| --- | --- | --- | --- | --- | --- | --- |
| ROV of Pp | 0.55 | 1.00 |  |  |  |  |
| ROV of Tm | 0.59 | -0.05 | 1.00 |  |  |  |
| CV of Pp | 0.52 | 0.84 | 0.12 | 1.00 |  |  |
| CV of Elev | 0.35 | 0.47 | 0.04 | 0.31 | 1.00 |  |
| CV of Tm | 0.85 | 0.48 | 0.51 | 0.45 | 0.21 | 1.00 |
| **One degree** | ROV of Elev | ROV of Pp | ROV of Tm | CV of Pp | CV of Elev | CV of Tm |
| ROV of Pp | 0.57 | 1.00 |  |  |  |  |
| ROV of Tm | 0.55 | -0.01 | 1.00 |  |  |  |
| CV of Pp | 0.61 | 0.67 | 0.32 | 1.00 |  |  |
| CV of Elev | 0.19 | 0.55 | -0.09 | 0.24 | 1.00 |  |
| CV of Tm | 0.86 | 0.49 | 0.46 | 0.54 | 0.07 | 1.00 |
| **Half degree** | ROV of Elev | ROV of Pp | ROV of Tm | CV of Pp | CV of Elev | CV of Tm |
| ROV of Pp | 0.46 | 1.00 |  |  |  |  |
| ROV of Tm | 0.53 | -0.09 | 1.00 |  |  |  |
| CV of Pp | 0.47 | 0.64 | 0.21 | 1.00 |  |  |
| CV of Elev | 0.08 | 0.24 | -0.17 | 0.18 | 1.00 |  |
| CV of Tm | 0.92 | 0.46 | 0.42 | 0.49 | 0.02 | 1.00 |
| **Quarter degree** | ROV of Elev | ROV of Pp | ROV of Tm | CV of Elev | CV of Pp | CV of Tm |
| ROV of Pp | 0.47 | 1.00 |  |  |  |  |
| ROV of Tm | 0.40 | -0.15 | 1.00 |  |  |  |
| CV of Elev | 0.02 | 0.23 | -0.20 | 1.00 |  |  |
| CV of Pp | 0.49 | 0.58 | 0.22 | 0.17 | 1.00 |  |
| CV of Tm | 0.93 | 0.48 | 0.32 | -0.03 | 0.51 | 1.00 |

**References**

1. Hijmans RJ, Cameron SE, Parra JL, Jones PG, Jarvis A. Very high resolution interpolated climate surfaces for global land areas. Int J Climatol. 2005;25(15):1965–78.

2. US Geological Survey. USGS Geospatial data clearinghouse, national mapping and remotely sensed data: digital elevation models (DEMs). 2010. p. https://lta.cr.usgs.gov/GMTED2010. Accessed 2011.

3. INEGI. Guia para la interpretación de cartografía uso del suelo y vegetación : Escala 1:250 000 : Serie III. Inegi [Internet]. 2009;77. Available from: http://sedico.campeche.gob.mx/Volumes/archivos/diabetes0.pdf

4. INIFAP–CONABIO. Edafología. Escalas 1:250000 and 1:1000000. Mexico City: Instituto Nacional de Investigaciones Forestales y Agropecuarias – Comisión Nacional para el Conocimiento y Uso de la Biodiversidad; 1995.
